# Effect of Substrate Etching on Terahertz Metamaterial Resonances and Its Liquid Sensing Applications

**DOI:** 10.3390/s20113133

**Published:** 2020-06-01

**Authors:** Sae June Park, John Cunningham

**Affiliations:** School of Electronic and Electrical Engineering, University of Leeds, Woodhouse Lane, Leeds LS2 9JT, UK; s.park@leeds.ac.uk

**Keywords:** substrate effect, metamaterials, liquid sensor

## Abstract

We investigate the effect of substrate etching on terahertz frequency range metamaterials using finite-element method simulations. A blue shift was found in the metamaterial resonance with increasing substrate etch depth, caused by a decrease in the effective refractive index. The relative contribution of the substrate’s refractive index to the effective refractive index was obtained as a function of the etch depth, finding that the decay length of the electric field magnitude below the *LC* gap is larger for the etched metamaterials due to their lower effective refractive index. We suggest designs for a terahertz metamaterial liquid sensor utilizing substrate etching which shows a significant enhancement in sensitivity compared to unetched sensors using ethanol as an example analyte. The sensitivity of the liquid sensor was enhanced by up to ~6.7-fold, from 76.4 to 514.5 GHz/RIU, for an ethanol liquid layer with a thickness of 60 μm by the incorporation of a substrate etch depth of 30 µm. Since the region of space close to the metamaterial is the most sensitive, however, we find that for small liquid thicknesses, larger etch depths can act to *decrease* sensitivity, and provide quantitative estimates of this effect.

## 1. Introduction

Metamaterials are structures designed to resonate with electromagnetic waves over a specific range of frequency controlled by their geometry and adjacent dielectric environment [1,2,3]. The inductive-capacitive (*LC*) resonance in metamaterials has received particular attention in the context of dielectric sensing applications owing to its high quality factor [4,5], and high sensitivity [6] compared to other resonances such as dipole and quadrupole modes. The *LC* resonant frequency of free-standing split-ring resonators (SRRs) is described by $f_{0}=1/2\pi \sqrt{LC}\;$, where *C* is the capacitance of the gap and *L* is the inductance of the ring [7,8]. However, the resonant frequency of metamaterials is not only determined by their geometry, but also by the effective refractive index (*n*_eff_) of the surrounding medium [9,10]. The *LC* resonant frequency *f* of an SRR located on a substrate can be described by *f* = *f*_0_/*n*_eff_, where *n*_eff_ can be expressed by the combined contributions of the substrate’s refractive index (*n*_sub_) and the air refractive index (*n*_air_) [10]. In the last decade, various improvements to terahertz (THz) metamaterial structures have been explored to optimize their use as dielectric sensors, such as use of low refractive index [11], and ultra-thin substrates [12], and the use of toroidal unit cells [13,14,15], as well as nano-gap structures [16]. It is noteworthy that ultrasensitive detection of the ZIKA virus with a limit of detection of 24 pg/mL has been demonstrated previously using magnetoplasmonic toroidal metamaterials [15]. Further, metamaterials fabricated on thin polyimide substrates have been proposed to demonstrate sensing of analytes from the top and bottom surfaces of the substrate, permitting dual-surface sensing [17]. We note, however, that thin substrates can be problematic in liquid sensing devices where the fluidic channels are incorporated due to their low inherent durability [18].

On the other hand, substrate etching is another such approach that can increase the sensitivity, aiming at reducing the effective refractive index near the metamaterial but partial removal of the substrate material. We recently demonstrated enhanced sensitivity in dielectric sensing applications by the introduction of *localized* etching confined to the active area of the metamaterial THz sensors [19]. This localized etching of the *LC* gap area was shown to provide significant enhancement of the sensitivity for cases where the on-resonance confined electric field is the strongest in the gap. However, the effect of etching of the surrounding substrate material close to the entire metamaterial geometry on metamaterial resonances has not yet been explored. Studying the contribution of the substrate on the effective refractive index as a function of etch depth is crucial to a full understanding of the effect on dielectric sensing. In this work, we present finite-element calculations of the *LC* resonant frequency of THz split-ring resonators for various substrate etch depths under conditions of dielectric loading by liquids, where the liquid fills the etched region. We consider here a case where the entire substrate around the metamaterial unit cell is etched to reduce the effective refractive index, rather than just the *LC* gap area. The relation between the effective refractive index and the substrate’s refractive index was obtained and is shown to vary significantly with the substrate etch depth. Investigation of the effect of substrate etching on the effective refractive index allows us to propose design considerations for optimized etched THz metamaterials in liquid sensing applications, with a predicted sensitivity enhancement factor of ~6.7 compared to unetched metamaterials.

## 2. Simulation Methods

**Figure 1:**
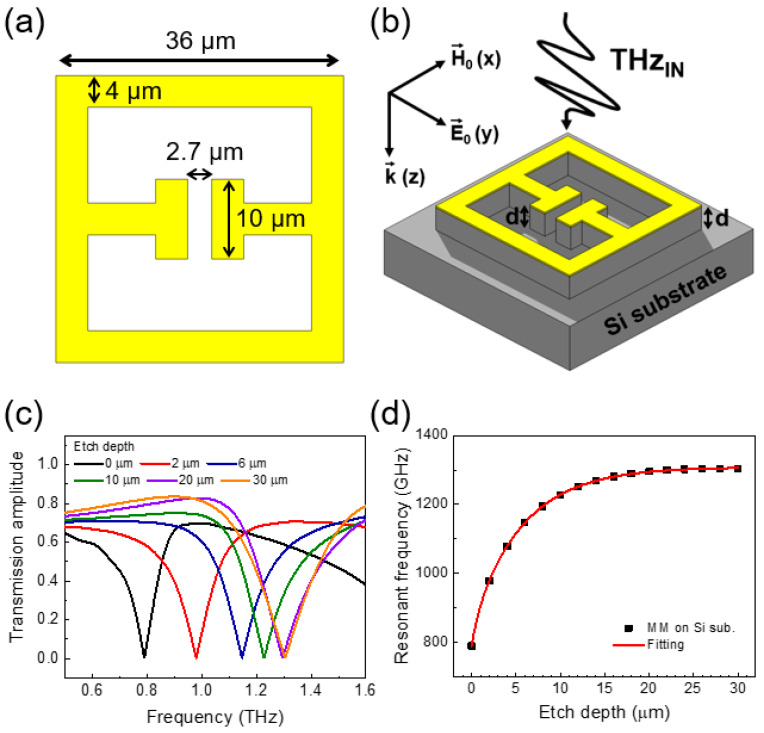
(**a**) Split-ring resonator (SRR) pattern used in the simulations. (**b**) Schematic of THz transmission simulation geometry used. Etch depth is labelled *d*, with linear THz polarization assumed in the y–z plane as shown. (**c**) THz transmission of the metamaterial for various etch depths around the fundamental resonance. (**d**) The resonant frequency of the metamaterial plotted as a function of etch depth *d*.

Our THz metamaterials were simulated using ANSYS High-Frequency Structure Simulator (HFSS) to calculate the frequency dependent simulated transmission and thereby resonant frequency. Figure 1a shows that the SRR consists of a rectangle with outer dimensions of 36 × 36 μm^2^, with a gap of 2.7 μm and a length of 10 μm introduced along one edge. The width and the thickness of the SRR were chosen to be 4 μm and 100 nm, respectively [19]. In order to obtain the transmission spectra (S_21_) of metamaterials, two-port S-parameter simulations were performed with a linearly polarized incident THz plane wave. Periodic boundary conditions with a periodicity of 50 µm around the unit cell were chosen, providing a similar areal density of the metamaterial elements to previous studies [20]. The transmission spectra obtained through the simulations showed good agreement in both the resonant frequency and quality factor to resonances found experimentally in our previous work [19]. A refractive index of 3.44 was used for Si substrate, as obtained using conventional THz time-domain spectroscopy methods in our prior work [19]. We note that an experimental error of ±0.02 for the Si substrate refractive index, which can induce knock-on changes of ~0.5% in the calculated resonant frequency of the metamaterials, was neglected here for simplicity. Figure 1b shows a schematic diagram of the metamaterial devices with etched substrate along with the polarization direction of the incident THz waves in the transverse-magnetic polarization geometry studied. Substrate etching was simulated by progressively removing the top surface of the substrate to an etch depth *d*, which was applied to the whole surface except where the metamaterial geometry was located (the metal features then effectively forming an etch mask in practical implementations).

## 3. Simulation Results and Discussions

Figure 1c shows the simulated transmission spectra for various substrate etch depths. A blue shift was found in the *LC* resonant frequency as the substrate etch depth was increased from 0 to 30 μm, caused by a progressive decrease in the effective refractive index near the metamaterial. Figure 1d shows the resonant frequency as a function of etch depth. The resonant frequency increases with increasing etch depth until saturation. This behaviour is caused by the electric field distribution of the metamaterials at the resonant frequency being mainly confined to the spatial region near the metamaterial elements [20], which will be discussed in detail later. The substrate contribution towards the effective refractive index is larger when the etch depth is small owing to this confinement. Hence, the blue shift in the resonant frequency is larger when the etch depth is relatively low, and reduces as the etch depth increases. We also note that we can estimate how effective our whole-substrate etching approach is at reducing *n*_eff_ by comparing the initial slope of the *f*–*d* curve in the linear region (*d* < 1 μm) in Figure 1d to that of a previously demonstrated localized etching approach [19]. We obtained a resonant frequency change rate of 115 GHz/μm for the whole-substrate etching proposed here, while 51 GHz/μm was obtained from the localized etching method previously investigated [19].

**Figure 2:**
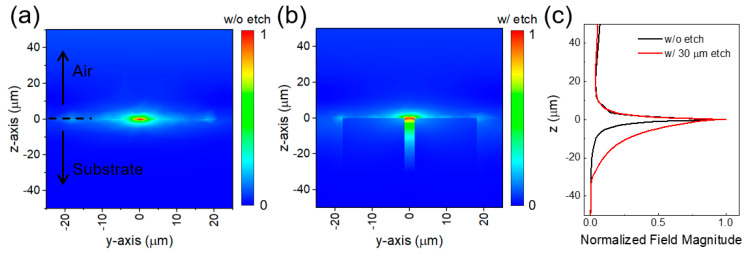
Field distribution near the metasurface at x = 0 for (**a**) without and (**b**) with an etch depth of 30 µm. (**c**) The electric field line profile along the z axis at x = 0 and y = 0 without (black line), and with (red line) an etch depth of 30 µm.

In order to understand the effect of the etching on the resonances further, the magnitude of the electric field near the metamaterial structures both with and without etching were investigated at the resonant frequency. Here, using the coordinate system shown in Figure 1a and with the centre of the metamaterial gap structure then located at x = 0, y = 0, and z = 0, the 2D electric field near the metamaterial structure along the y–z plane at x = 0 both without and with etching (etch depth of 30 µm) was calculated, as shown in Figure 2a,b respectively. We note that the electric field distribution shown in Figure 2b extends further towards the etched substrate surface. This is due to the decrease in the effective refractive index underneath the metamaterial geometry upon etching. The electric field magnitude along the z axis at x = 0 and y = 0 was extracted from Figure 2a,b and plotted in Figure 2c. The electric field magnitude at the resonant frequency has a maximum value at the centre of the gap structure and then decays exponentially towards both the substrate and the air [19,20]. The decay length of the electric field magnitude obtained by an exponential fit below the gap structure is increased approximately ~4-fold (from 2.2 to 8.7 µm) for an etch depth of 30 µm. These results indicate that etch depths larger than 30 µm will be not effective in reducing the effective refractive index for typical THz metamaterial geometries similar to those discussed here.

**Figure 3:**
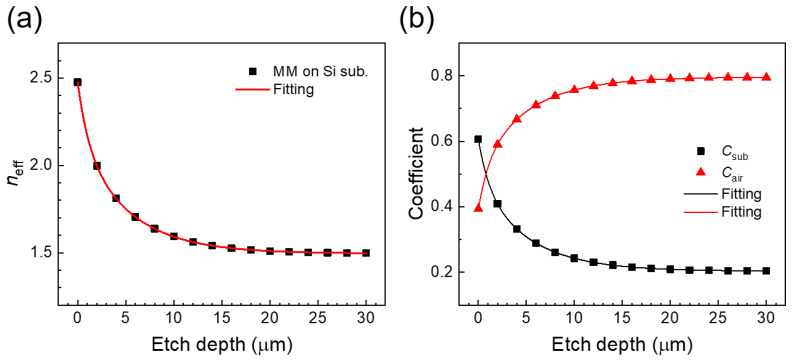
(**a**) *n*_eff_ as a function of substrate etch depth. (**b**) A plot of coefficient *C*_sub_ and *C*_air_ as a function of substrate etch depth.

In Figure 3a, the effective refractive indices near the metamaterials were extracted from the resonances shown in Figure 1c using the *f* = *f*_0_/*n*_eff_. *n*_eff_ saturates exponentially with etch depth, which can be explained by the electric field distribution near the metamaterials reaching down to 30 µm from the air–substrate interface in *z* direction, as shown in Figure 2c [19,20]. We were able to significantly reduce the effective refractive index near the metamaterial from ~2.47 to ~1.49 by the introduction of the 30 µm etch. We note that this reduction in effective refractive index to ~1.49 is lower than the effective refractive index achieved by replacing the substrate materials with a quartz substrate (*n*_eff_ ~ 1.6) [9].

We investigate the relative contributions of the substrate and the air on the effective refractive index, based on the model function *n*_eff_ = *C*_sub_*n*_sub_ + *C*_air_*n*_air_ [10], where *C*_sub_ and *C*_air_ are the substrate and air co-efficient contributions, respectively. *C*_sub_ and *C*_air_ were extracted from Figure 3a in Figure 3b. The contribution from the substrate on the effective refractive index near the metamaterials was found to be ~61% (*C*_sub_ ~ 0.61) without etching, but this was reduced to ~20% (*C*_sub_ ~ 0.20) by introducing a substrate etch depth of 30 µm, for example. For dielectric sensing using the metamaterial, this change in the effective refractive index can dramatically improve the sensitivity, as discussed later. It is also noteworthy that we can also increase the interaction volume between the dielectric and the metamaterials when the etched region can be filled by a dielectric. This method is, therefore, particularly suited to liquid sensing, since we can then take advantage both of the reduced effective refractive index and the increased interaction volume. The sensitivity enhancement achieved depends on the etch depth and the thickness of the liquid, however, as explored below.

**Figure 4:**
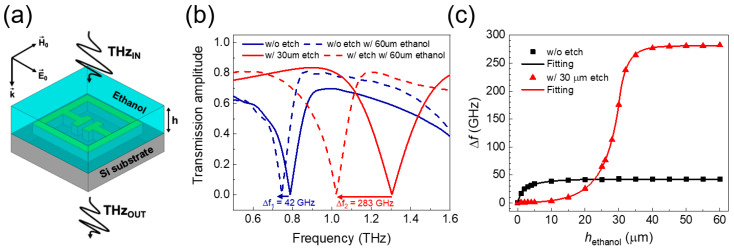
(**a**) Schematic of the THz metamaterial device with substrate etch for sensitive liquid sensing. (**b**) THz transmission of the metamaterials both with (red lines) and without (blue lines) an etch depth of 30 µm, and both with (dashed lines) and without (solid lines) the presence of an ethanol liquid layer with a thickness of 60 μm. (**c**) Resonant frequency shift as a function of *h*_ethanol_ for the metamaterial liquid sensors with (red triangles) and without (black squares) an etch depth of 30 µm.

To study the effect of the substrate etch on the sensitivity for liquid sensing applications, we investigated the enhancement in sensitivity for varying thicknesses of a liquid layer deposited onto the etched metamaterial, as shown in Figure 4a. Ethanol was chosen as the analyte, since it is a crucial material in several industrial contexts, including its use in the beverage industry, where developing ethanol sensors is a requirement to the determine alcohol content of drinks [21]. Figure 4b shows the simulated THz transmission of the metamaterial both with and without a substrate etch depth of 30 µm, and both with and without the deposition of an ethanol liquid layer with a thickness of 60 μm. The ethanol layer was assumed to have a THz refractive index of 1.55 (= *n*_ethanol_), as previously measured at 0.8 THz in previous work [22]. We used an etch depth of 30 μm, since the effective refractive index near the metamaterial is already saturated for this depth, as shown in Figure 3a. A liquid layer thickness (*h*_ethanol_) of 60 μm was chosen to compare the fully saturated resonant frequency shift for both etched and unetched devices. The size of the resonant frequency shift obtained after deposition of an ethanol liquid layer with a thickness of 60 μm increased by a factor of ~6.7 upon etching, from 42 to 283 GHz.

Finally, the resonant frequency shift of the sensors was compared both with and without a substrate etch depth of 30 µm as a function of liquid thickness (with the latter measured from the bottom of the etched region), as shown in Figure 4c. The resonant frequency changes significantly, at a rate of 46 GHz/µm for the etched device, and 24 GHz/µm for the unetched device when the liquid layer is close to the metal of the metamaterial (*z* ~ 0 in Figure 2c). Therefore, devices without etch are most sensitive near *h*_ethanol_ = 0 µm, while devices with 30 µm etch are most sensitive for a thickness of *h*_ethanol_ = 30 µm. We, therefore, find that, for small liquid thicknesses, larger etch depths act to *decrease* the size of the resonant frequency shift. For example, devices without substrate etching show a resonant frequency shift of 17 GHz for *h*_ethanol_ = 1 µm. Conversely, devices with an etch depth of 30 µm show a resonant frequency shift of 860 MHz for *h*_ethanol_ = 1 µm.

The saturated frequency shift increases as the etch depth is increased owing to the decrease in the effective refractive index and increased interaction volume. From Figure 4c, the sensitivities were obtained by dividing the saturated resonant frequency shift by the refractive index change (Δ*n* = *n*_ethanol_ − *n*_air_). The sensitivity (S) of the metamaterial liquid sensor increased from 76.4 (without etch) to 514.5 GHz/RIU (with a 30 μm etch), an increase of ~6.7-fold, for an ethanol liquid layer with a thickness of 60 μm. We note that the sensitivity obtained was rather higher than that obtained using a low-permittivity substrate (250 GHz/RIU) [23]. Table 1 shows a comparison of the sensitivity in this work to others recently demonstrated. The figure-of-merit (FOM) values were enhanced from 0.8 without etch to 3.4 with a 30 μm etch, as calculated using the following relation: FOM = S(in nm/RIU unit)/FWHM(in nm unit), where FWHM is the full width at half maximum of the resonance without the analyte.

## 4. Conclusions

We investigated the contribution of the substrate refractive index to the resonant frequency of THz metamaterials when substrate etching was introduced. The *LC* resonant frequency of metamaterials was studied for various etch depths on Si substrates. A blue shift was found as etch depth increased up to 30 µm. The electric field distribution near the metasurface was simulated for both with and without etching to understand the effect of etching at the resonant frequency. We also obtained the relation between the effective refractive index near the metamaterial and the substrate refractive index for various etch depths. We were able to reduce the substrate’s contribution down to 20% by the introduction of a 30 µm substrate etch. Substrate etching could be used to develop a sensitive liquid sensor in the THz frequency range. By reducing the effective refractive index and increasing the interaction volume between the metamaterial and the liquid, a sensitivity of 514.5 GHz/RIU was obtained with an etch depth of 30 µm for an ethanol liquid layer with a thickness of 60 μm, which is 6.7-fold more sensitive than the device without etch. Conversely, our study also reveals that a substrate etching approach can decrease the sensor sensitivity for deep etches with thin layers of liquid, for example resulting in a reduction in the sensitivity from 30.5 (without etch) to 1.6 GHz/RIU (with 30 µm etch) for *h*_ethanol_ = 1 µm.

## Figures and Tables

**Table 1:** Performance of recently demonstrated THz metamaterial dielectric sensors. RIU sensitivities are presented in two units. GHz/nm for the sensors that are focusing on the surface sensing near the metamaterials where the sensitivity depends on the analyte thickness, and GHz for the sensors using the saturated resonant frequency shift for its sensing where the sensitivity is independent on the analyte thickness.

Structure Type	Substrate Type	RIU Sensitivity	References
Asymmetric SRR	25 µm thick cyclic olefin copolymer	1 GHz/nm	[24]
Double SRR	1 µm thick silicon	0.07 GHz/nm	[25]
SRR with localized substrate etching	500 µm thick silicon	0.25 GHz/nm	[19]
Toroidal SRR	25 µm thick mylar	186 GHz	[13]
SRR with substrate etching	500 µm thick silicon	515 GHz	This work
